# The relationship between food selectivity and stature in pediatric patients with avoidant-restrictive food intake disorder – an electronic medical record review

**DOI:** 10.1186/s40337-024-01020-0

**Published:** 2024-05-21

**Authors:** Kaitlin B. Proctor, Maryam Mansoura, Eugene Rodrick, Valerie Volkert, William G. Sharp, Joseph M. Kindler

**Affiliations:** 1 Division of Autism & Related Disorders, Department of Pediatrics, Emory University School of Medicine, Atlanta, GA USA; 2 Children’s Healthcare of Atlanta, Atlanta, GA USA; 3 Medical College of Georgia, Augusta University, University of Georgia Medical Partnership, Augusta, GA USA; 4 Department of Nutritional Sciences, University of Georgia, Athens, GA USA

**Keywords:** Avoidant/restrictive food intake disorder, Stature, Dietary intake

## Abstract

**Background:**

We aimed to characterize stature in pediatric patients with avoidant/restrictive food intake disorder (ARFID), including associations between body size and nutrient intake and height.

**Methods:**

We conducted a secondary analysis of pre-treatment data from 60 patients diagnosed with ARFID that were collected from the electronic medical record. Anthropometric measurements were converted to age- and sex-specific Z-scores using pediatric CDC growth charts. Spearman correlations were performed to test the relationship between height and weight/BMI Z-scores as well as height Z-score and diet variables.

**Results:**

On average, height (-0.35 ± 1.38), weight (-0.58 ± 1.56), and BMI (-0.56 ± 1.48) Z-scores tended to be lower than what would be expected in a generally healthy pediatric population. Percent of individuals with height, weight, or BMI Z-score < -2.0 was 8%, 20%, and 17%, respectively. BMI (*P* < 0.05) and weight (*P* < 0.05) were positively associated with height Z-score. Further, intake of some nutrients (e.g., calcium, vitamin D) correlated positively with height Z-score (all *P* < 0.05).

**Conclusions:**

The cross-sectional relationships reported in this study suggest that in children with ARFID, body weight and consumption of bone-augmenting nutrients such as calcium and vitamin D correlated with height. A thorough understanding of the clinical manifestations of malnutrition and longitudinal effects of restrictive eating in patients with ARFID is critical.

## Abstract (plain language summary)

We examined data on growth and height for a sample of 60 children with highly selective eating consistent with an eating/feeding disorder termed avoidant/restrictive food intake disorder (ARFID). These children received treatment in an intensive multidisciplinary intervention program. We found that children had significantly lower weight and body mass index (BMI) compared to same sex and age peers, with a trend toward lower height. Greater body size and intake of specific nutrients was related to taller stature in this sample. Children with ARFID may be at greater risk of impaired growth secondary to highly restricted food intake, a health outcome which should be studied to inform screening and intervention practices.

## Introduction

Avoidant/restrictive food intake disorder (ARFID) is a condition in the Diagnostic and Statistical Manual of Mental Disorders, Fifth Edition, Text Revision (DSM-5-TR) describing patients with severely limited dietary intake absent of body image disturbances or body weight concerns [1]. The key features of ARFID include one or more of the following: weight loss or impaired growth (A1), nutritional deficiencies (A2), reliance on nutritional supplements or formula (oral and/or enteral administration; A3), and/or interference with psychosocial functioning (A4) [1–4]. Nutritional status in patients with ARFID is highly variable, but the diet of the A2 subtype has generally been characterized as being high in processed foods and added sugars and low in protein and vegetables [5]. At present, little is known about the impact of highly restrictive food intake characteristic of ARFID on longitudinal growth. There is extensive research in pediatric patients with anorexia nervosa (AN) indicating that malnutrition from restricted intake, particularly when co-occurring with low BMI, is associated with deficits in longitudinal growth and bone accrual [6, 7]. Growth in stature and bone accrual might also be threatened in ARFID [8, 9], but there is need for additional research given that ARFID typically has an earlier onset and a more chronic course compared to AN [6, 8, 10–12]. 

This study was an exploratory analysis of data from a retrospective chart review describing pre-treatment outcomes for children with severe food selectivity participating in an intensive multidisciplinary intervention (IMI) program. The main objective of the present study was to describe the pre-treatment physical characteristics of pediatric ARFID patients, particularly height, which was not reported in the original study [13]. We also examined associations between height and indicators of nutritional status and diet. We hypothesized that patients with ARFID would have shorter stature compared to pediatric normative growth curves, which would be associated with smaller body size.

## Methods

### Participants and setting

We conducted a secondary analysis of data from pediatric patients with ARFID [13]. Data were retrieved from the electronic medical record (EMR), representing a cohort of consecutive patients (birth to 21 years of age) treated in an IMI pediatric feeding disorder program from June 2014 to June 2019 located in the southeastern United States. For inclusion, patients were required to (1) meet criteria for the A2 ARFID subtype based on micronutrient insufficiencies associated with severe food selectivity, (2) engage in refusal behaviors during feeding necessitating intensive intervention, and (3) be medically cleared to consume new foods. ARFID was diagnosed by a multidisciplinary assessment team that included medical, nutrition, psychology, and feeding skill providers. Micronutrient insufficiencies were determined by registered dietitian (RD) dietary assessment. Comorbid conditions other than ARFID that were listed in the medical chart were reviewed, and parents were asked about any additional medical, developmental, or behavioral conditions during clinical interviews.

We elected to restrict our sample in the present study to children presenting with primary concern for nutritional insufficiencies with or without low weight, as these children demonstrated a prolonged, systematic avoidance of specific types of food. Children presenting predominately for concerns related to poor growth/weight loss were excluded, as these patients eat an insufficient volume but may have a wider variety of foods accepted. Children dependent on oral or enteral nutritional supplements were also excluded, as they are likely to have fuller nutritional coverage from supplementation. Children with marked interference in psychosocial functioning without documented health impacts or formula dependence were also excluded, as these children were not yet exhibiting immediate health impacts due to restrictive eating. Children who demonstrated severe, problematic behaviors such as aggression and/or self-injury were also excluded from analyses, as these children required a different intervention to address these behaviors prior to feeding intervention. Only data from the time of admission to the IMI program were used for this study.

### Anthropometrics

Height and weight were measured using a digital scale and wall-mounted stadiometer, respectively. Centers for Disease Control and Prevention pediatric growth charts were used to calculate Z-scores for height, weight, and BMI.

### Diet assessment

Dietary intake was assessed using a multimethod approach. A registered dietitian (RD) met with each patient’s family to obtain a list of foods the child consistently accepted, as well as to conduct a dietary recall interview reflective of the child’s typical intake. The RD analyzed the dietary recall using Food Processor Pro 14.10x to determine average intake of micronutrients, macronutrients, energy, and food groups. Nutrient values were expressed relative to DRI [14, 15]. 

### Statistical analysis

Data were summarized as mean/standard deviation and count/percentage for continuous and grouped variables, respectively. Spearman rank correlation was used to test the relationship between height, weight, and BMI Z-scores, and the relationship between nutritional status and diet variables and height Z-score. Relationships between diet variables and height Z-score were also performed while including only individuals with a BMI-for-age greater than the 5th percentile, therefore excluding those with “underweight.” Sensitivity analyses were performed while excluding potential outliers/influential data points from the dataset. Analyses were performed using STATA version 15. *P*-values less than 0.05 were considered statistically significant.

## Results

Sample characteristics were reported previously [13]. One hundred fifty-seven children were excluded from analysis due to impaired growth, formula reliance, psychosocial impairment only without immediate medical sequela, or presence of severe behavior. Our sample included 60 children ages 1.9 to 15.1 years (Table 1). The majority were male (83%) and identified as non-Hispanic (95%) and White/Caucasian (58%) or Black/African American (30%). Most patients (95%) presented with one or more medical and/or developmental conditions, with 80% reporting multiple medical/developmental diagnoses. In this sample, autism spectrum disorders were the most common comorbid condition (63%), followed by gastroesophageal reflux disease (40%) and constipation (38%).

**Table 1:** Demographic characteristics (*N* = 60)

Characteristic	Mean (SD)	Range
Age (months)	72 *(39.0)*	23–181
Anthropometrics (Z-scores)		
Height	-0.35 *(1.38)*	-5.36–2.20
Weight	-0.58 *(1.56)*	-4.94–2.28
BMI	-0.56 *(1.48)*	-4.60–2.33
	n (Percent)	
Sex		
Male	50 (83.3)	
Female	10 (16.7)	
Race		
White/Caucasian	35 (58.3)	
Black/African American	18 (30.0)	
Asian	6 (10.0)	
American Indian/Alaskan Native	1 (1.7)	
Ethnicity		
Hispanic	2 (3.3)	
Non-Hispanic	57 (95.0)	
Missing/Not Reported	1 (1.7)	

Height (-0.35 ± 1.38), weight (-0.58 ± 1.56), and BMI (-0.56 ± 1.48) Z-scores in the ARFID sample were less than 0 on average but were highly variable. Height Z-score ranged from − 5.3 to 2.2, weight Z-score ranged from − 4.9 to 2.3, and BMI Z-score ranged from − 4.6 to 2.3. The percent of the ARFID sample with a height, weight, or BMI Z-score < -2.0 was about 8%, 20%, and 17%, respectively.

**Fig. 1:**
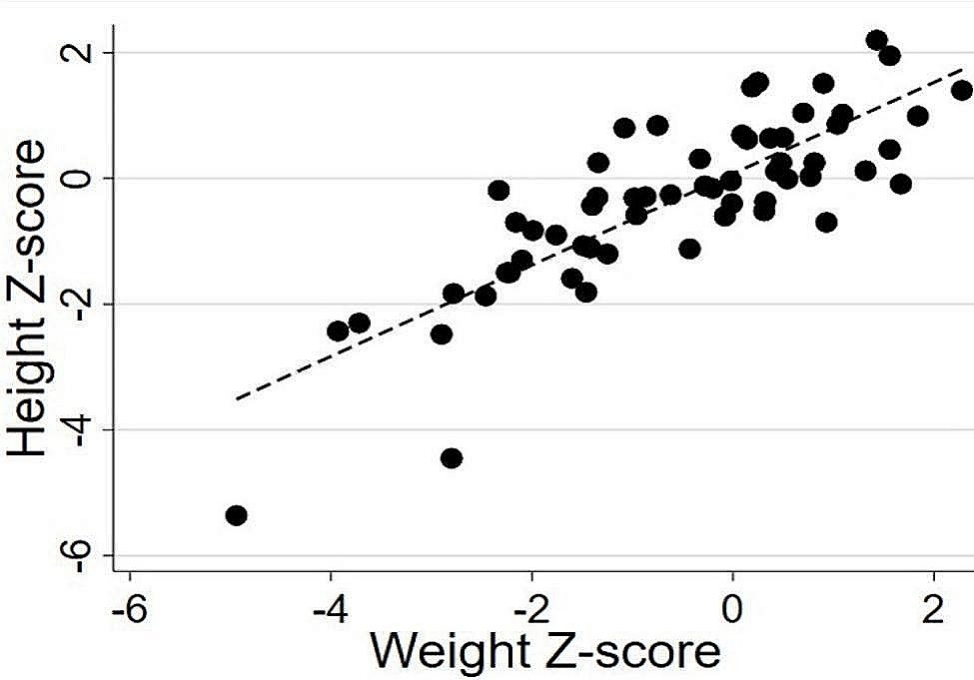
Spearman correlations between weight and height Z-scores

**Fig. 2:**
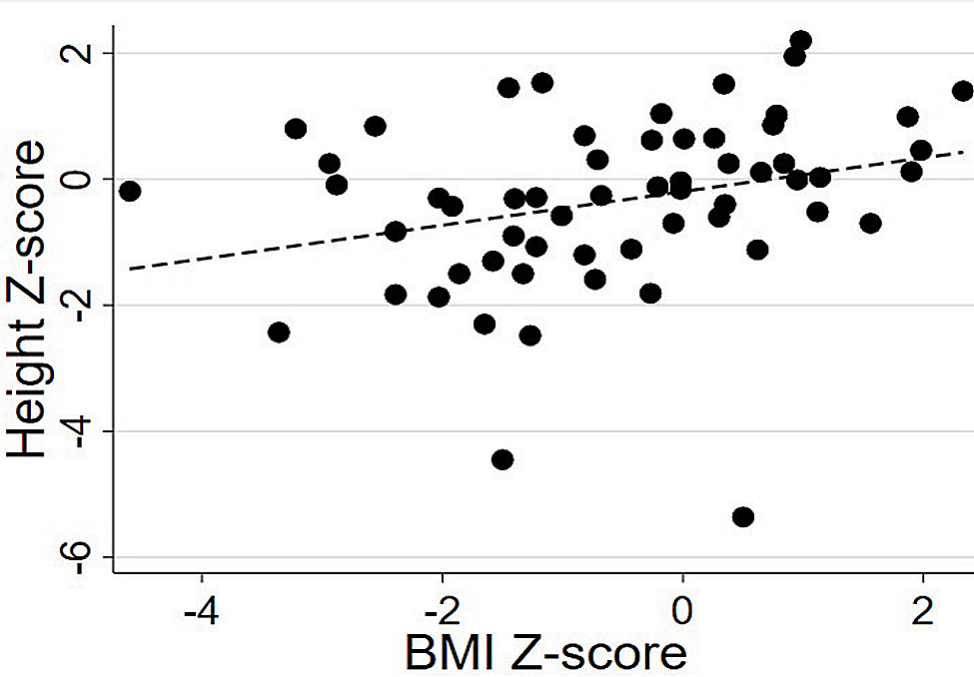
Spearman correlations between BMI and height Z-scores

Bivariate correlations between weight and BMI Z-scores with height Z-score are presented in Figs. 1 and 2, respectively.

Weight (rho = 0.802, *P* < 0.001) and BMI (rho = 0.387, *P* < 0.005) Z-scores were positively correlated with height Z-score, such that individuals with lower body weight/BMI tended to be shorter compared to those with greater body weight/BMI. Vitamin A, vitamin B12, vitamin D, folic acid, and calcium were positively associated with height Z-score (all *P* < 0.05; Table 2). When excluding individuals with a BMI-for-age less than the 5th percentile, the significant associations between vitamin B12, vitamin D, and calcium remained statistically significant (all *P* < 0.05). Age did not correlate with height, weight, or BMI Z-scores (rho = -0.10 to 0.08, *P* = 0.43 to 0.59). All analyses were rerun while excluding the two individuals with height Z-score < -4.0. All associations described above were maintained (results not presented in detail).

**Table 2:** Spearman correlations between diet variables and height Z-score

	Rho	*P*
Total kcal	0.15	0.246
Vitamin A	0.30	0.022*
Vitamin B12	0.41	0.003*
Vitamin C	0.00	0.983
Vitamin D	0.42	0.001*
Vitamin E	0.05	0.734
Folic acid	0.30	0.026*
Calcium	0.34	0.010*
Iron	0.14	0.323
Zinc	0.16	0.266

*p < 0.05

## Discussion

Eating/feeding disorders have been linked with growth disturbances and likely have a profound impact on longitudinal growth and bone mass acquisition [6–8]. ARFID is relatively new in the diagnostic nomenclature and research on the impact of food restriction in this population has been sparse. The etiology of ARFID differs from that of more extensively researched restrictive food intake disorders such as AN; thus, there is a need for studies focused specifically on youth with ARFID to determine the extent to which growth is impacted [2, 3, 8–12]. The current study tested the association between ARFID (A2 subtype) and stature in pediatric patients. The incidence of comorbid medical and developmental conditions in our sample was high, which is expected given that an estimated 40–80% of children with medical/developmental conditions evidence feeding concerns [16]. Although highly variable, patients with ARFID tended to have shorter stature and smaller body size compared to what would be expected in the general healthy pediatric population. A secondary objective was to assess relationships between body size and dietary intake with height Z-score. We found that smaller body size, as indicated by a lower weight and/or BMI Z-score, as well as lower intake of several key nutrients in musculoskeletal development, calcium and vitamin D, were inversely correlated with height Z-score. Results from this small, cross-sectional investigation of medical record data support the limited data available in the literature and affirms the need for rigorous research involving growth in pediatric patients with ARFID [6, 8, 9]. 

Longitudinal growth is reliant on appropriate nutritional status. Growth deficits in youth with eating disorders, such as AN, are well documented; [6] however, there is a need for studies focused specifically on the ARFID patient population [8]. We found that children with ARFID had slightly shorter stature compared to CDC pediatric growth reference curves, with an average height Z-score of -0.35. This translates to ARFID patients having, on average, an approximately one-third SD lower height compared to normally developing youth, since it would be expected that a sample of children drawn from the general healthy population would have a mean Z-score of 0. Based on a standard normal distribution, it is expected that about 2–3% of healthy children would have a height Z-score less than − 2.0. In this study, 8% of patients met or exceeded this value. Similar findings have been reported in the UK by Alberts and colleagues, where pediatric ARFID patients, ages 6–19 years, had shorter stature compared to a healthy UK reference dataset [6]. It should be noted that the criteria for ARFID classification employed in the current study differed from the criteria used by Alberts and colleagues, which only included patients who were underweight. Our sample focused specifically on individuals with the A2 ARFID subtype, without criteria for weight status. Even more, we excluded children presenting predominately for concerns related to poor growth/weight loss and those that were dependent on nutritional supplements. Thus, body size was more variable in our sample, with approximately 17% of patients having a BMI Z-score less than − 2.0. Furthermore, our correlation analyses revealed a strong association between body size and height, suggesting that individuals with a low body weight/BMI had a greater tendency towards shorter stature compared to those with larger body size. In the future, body weight screening may help identify youth with ARFID at greatest risk for growth deficits who would benefit from additional bone health assessment.

Poor nutritional status in children with ARFID might be accompanied by inadequate intake of key nutrients and food groups that augment longitudinal growth. In this study, vitamin A, vitamin B12, vitamin D, folic acid, and calcium were positively associated with height Z-score in children and adolescents with ARFID. Kim and colleauges [17] reported similar relationships between diet measures and height Z-scores in otherwise healthy children. Namely, height Z-score was positively associated with energy intake and consumption of vitamin A, vitamin D, vitamin B12, and calcium, among others. These nutrients have been studied as potential biomarkers for growth and development and may have diffuse impacts on various health-related outcomes [18]. For example, in children with vitamin A deficiency, supplementation helps prevent infection and diarrhea, thereby promoting growth and development [19]. Vitamin B12 and folate are important for erythropoiesis, homocysteine metabolism, and cognitive and neurologic function [20]. In patients who are clinically deficient, supplementation with these nutrients is used to treat megaloblastic and pernicious anemia, the latter of which can lead to osteoporosis and impaired growth [20]. These diet factors might contribute to growth deficits in children with restrictive food intake disorders such as ARFID, but it is important to interpret these associations in light of the limitations of self- and parental-reported diet intake in children and adolescents. Prior studies have shown modest effects of single and combined micronutrient interventions on child growth outcomes in toddlers and school-aged children with and without overall increase in caloric intake [21–24], although improved total caloric intake via supplementation or food fortification has been proposed as a confound to clear delineation for causation. Improved baseline nutritional status likely moderates the effect sizes observed for micronutrient supplementation not solely attributable to improved caloric intake [21, 22, 24]. Our cross-sectional study design precludes inferences of causality regarding involvement of diet and nutritional status longitudinal growth.

Vitamin D insufficiency has been reported as commonly occurring in youth with ARFID [25]. Vitamin D is critical in the regulation of several biological processes related to growth, including the facilitation of calcium absorption in the intestines and of bone turnover [25]. In the current study, both vitamin D and calcium intake were associated with height Z-score. The human skeleton serves as the primary storage reservoir for calcium in the form of hydroxyapatite, along with other minerals. Children with ARFID are prone to developing medical sequalae of vitamin D deficiency including rickets, which is associated with several musculoskeletal manifestations including under-mineralized skeleton, bowing of the legs, and bone pain [26, 27]. 

Impaired longitudinal growth and low body weight due to restrictive eating may have long-term health implications beyond the growing years. Children may experience comorbid psychosocial and cognitive disturbances because of malnutrition, including anxiety and depression, along with comorbid eating disorders [11, 28–30]. Moreover, malnutrition may have a substantial impact on physiologic functions, such as hormonal pathways (e.g., menarche and menstruation) and bone mass accrual [9]. It is well known that malnutrition interferes with hypothalamic pathways, causing amenorrhea due to lack of estrogen [18]. Additionally, estrogen also plays a central role in bone metabolism. Therefore, these patients may be at increased risk for low bone mineral density, which in turn increases the risk of fractures and osteoporosis [18]. Recent studies suggest that children and adults with ARFID have deficits in bone density that are similar to those observed in AN. Additional research is needed to determine the impacts of malnutrition in patients with ARFID on future health status, including attainment of peak bone mass and risk for fracture.

Limitations of this study included the use of cross-sectional data that were acquired from the EMR. Given the high rates of medical and developmental comorbidity within the sample, we cannot speak to the potential mediating role of medical conditions in the observed stature outcomes. Given the high rate of comorbid medical conditions expected within the ARFID population, this represents a significant need in future research to further elucidate the contribution of restrictive eating on growth outcomes independent of co-occurring medical conditions. Even more, impaired growth might also contribute to, or occur alongside, other developmental outcomes. A systematic review and meta-analysis of observational studies by Sudfeld and colleagues showed that linear growth is strongly associated with myriad cognitive and motor developmental outcomes in children [16]. Another limitation in this study was the use of parental reports for the assessment of dietary intake. This introduced potential recall bias and error.

While objective measures of serum vitamin D status and bone density data were not available in the current study, stature provides an important proxy of bone health signaling that undernutrition secondary to highly restrictive eating begins impacting child health during the growing years. An in-depth examination of bone health biomarkers will help understand the mechanisms of restrictive eating on child bone biology, particularly the degree to which compromised bone health and child growth can be remediated with intervention. Finally, although the scope of this study may seem limited due to the inclusion of only individuals with the ARFID A2 subtype, these findings represent a unique examination of an under-studied clinical cohort of children presenting with restricted intake. Many studies to date examining bone biology secondary to restrictive eating have focused solely on low-weight individuals but our sample was heterogenous with respect to body size. This paper expands the current literature by including children based on patterns of restrictive eating without exclusion based on body weight.

In conclusion, children with ARFID have restrictive eating habits that might impact growth. Our cross-sectional findings suggest that poor nutritional status in pediatric patients with ARFID may threaten longitudinal growth, but prospective studies are required to clarify these findings. Future studies should incorporate a larger sample of patients spanning all ARFID subtypes and comorbidities, have standardized timepoints for repeated measurement, and include biomarkers of dietary intake and nutritional status.

## Data Availability

The data that support the findings of this study are available from the corresponding author upon reasonable request.
